# Natural Occurrence of Ochratoxin A in Spices Marketed in the Czech Republic during 2019–2020

**DOI:** 10.3390/foods10122984

**Published:** 2021-12-03

**Authors:** Darina Pickova, Jakub Toman, Vladimir Ostry, Frantisek Malir

**Affiliations:** 1 Department of Biology, Faculty of Science, University of Hradec Kralove, Rokitanskeho 62, CZ-50003 Hradec Kralove, Czech Republic; ostry@chpr.szu.cz (V.O.); frantisek.malir@uhk.cz (F.M.); 2 Center for Health, Nutrition and Food in Brno, National Institute of Public Health in Prague, Palackeho 3a, CZ-61242 Brno, Czech Republic

**Keywords:** spices, ochratoxin A, immunoaffinity columns, HPLC-FLD

## Abstract

Spices are a popular ingredient in cuisine worldwide but can pose a health risk as they are prone to fungal infestation and mycotoxin contamination. The purpose of this study was to evaluate ochratoxin A (OTA) in 54 single-kind traditional and less traditional spices, each of which was purchased in six samples of different batches (324 samples in total) at the Czech market during 2019–2020. The HPLC-FLD method with pre-treatment by immunoaffinity columns was employed to determine OTA. The limits of detection and quantification were 0.03 ng g^−1^ and 0.10 ng g^−1^, respectively. A total of 101 (31%) samples of 19 spice kinds were positive at concentrations ranging from 0.11–38.46 ng g^−1^. Only turmeric was contaminated with an OTA level exceeding the European Union limits. However, most spices have no regulation, thus further extensive monitoring of various mycotoxins in various kinds of spices is necessary. Chilli and black pepper are the most studied spices for OTA contamination, however, many other kinds of spice can also be highly contaminated, but studies on them are less common, rare, or have not yet been performed. The uniqueness of this study lies in the wide range of spice types studied for the presence of OTA on the Czech market.

## 1. Introduction

There are several definitions for spices that may to some extent overlap with herbs [1,2,3]. Unlike herbs, which are defined as plants with non-woody tissues, spices are considered a culinary term rather than a botanical category [2]. This study is guided by the simple definition that spices are all parts of a plant that are used to improve meals in their colour, flavour, or even texture. These parts can be leaves, seeds, roots, fruits, bark, buds, or stalks [3].

The importance of spice may vary through countries worldwide [2]. Although generally thought to represent only a small portion of the human diet, they cannot be neglected as they may contribute to the overall intake of mycotoxins from all foodstuffs [4]. Spices are a widespread commodity [2] as they are exported worldwide, mainly from developing countries where they are mostly grown. Approximately 15.9 million tonnes of spices (excluding garlic and onion together exceeding 100 million tonnes) were produced in 2019 [5]. Asian countries were the largest producers of spices (share of production 75.7%; 12.1 million tonnes), followed by African (19.9%; 3.2 million tonnes), American (3.8%; 0.6 million tonnes), European (0.5%; 0.08 million tonnes), and Oceanian (0.1%; 0.012 million tonnes) producing countries in 2019.

Unfortunately, spices are susceptible to fungal infestation and mycotoxin contamination. The local subtropical/tropical climate conditions in most spice-producing countries such as high temperatures in combination with heavy rainfalls pose a suitable environment for mould infestation and thus mycotoxin production in spices. Moreover, following good agricultural, hygienic, and manufacturing practices is particularly difficult in these countries, which is also likely to contribute to the deterioration of spices by moulds and mycotoxins [6,7,8,9]. *Aspergillus carbonarius*, *A. flavus*, *A. ochraceus*, *A. parasiticus*, *A. tamarii*, *A. versicolor*, *Penicillium citrinum*, *P. verrucosum*, and *Fusarium verticillioides* are considered the most common moulds in spices. However, *Alternaria alternata*, *Rhizopus oryzae,* and *R. nigricans* have also been found in some spices such as cumin and coriander [4]. Ochratoxin A (OTA), along with aflatoxins B1, B2, G1, and G2, citrinin, fumonisins B1 and B2, trichothecenes such as deoxynivalenol, nivalenol, T-2 toxin, and HT-2 toxin, zearalenone, altenuene, alternariol, tenuazonic acid, and sterigmatocystin, has been confirmed in spices by many studies [4].

This study focused on OTA (PubChem CID: 442530) [10], which is considered the second most important mycotoxin from the public health point of view [11]. Moreover, it is infamous mainly for its nephrotoxic and less hepatotoxic effects, however, teratogenic, genotoxic, immunotoxic, and neurotoxic effects have also been reported [12]. The International Agency for Research on Cancer classifies OTA into group 2B, which is a possible carcinogen for humans [13,14].

A recent study [4] describing the situation of spice mycotoxin and mould contamination revealed that besides the well-known and most studied spices such as chilli and black pepper, many other types of spices also deserve attention and need to be monitored for various mycotoxins. Table 1 shows a summary of the results of recent studies on the determination of OTA in several types of spices [15,16,17,18,19,20,21,22,23,24,25,26,27,28,29,30,31,32,33,34,35,36,37,38,39,40,41,42,43,44,45,46].

However, several single-kind spices have never (or not recently) been tested for OTA. Therefore, this study aims to determine OTA in a wider range of spice types to obtain an overview of the current state of the OTA contamination of spices sold on the Czech market. As far as the authors know, globally, this is the first study dealing with OTA in many kinds of spices that are based on a single plant species.

## 2. Materials and Methods

### 2.1. Sample Collecting

**Figure 1:**
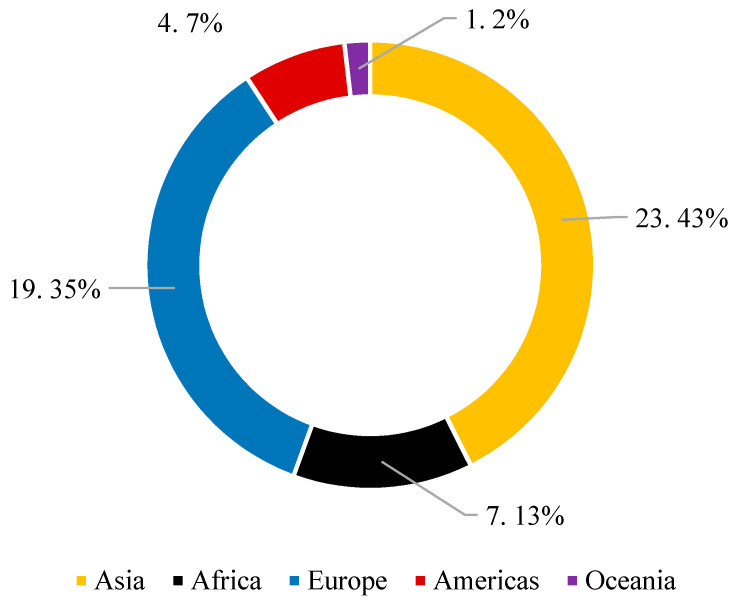
The origin of spice samples.

Fifty-four single-kind of traditional and less traditional spices (six samples of different batches per kind of spice, 324 samples in total, each in the amount of 30–100 g) were collected in the years 2019–2020. Of this number, 300 samples (92.6%) were imported and 24 samples (7.4%) were of Czech provenance. The characterisation of all single-kind spice samples was performed (see Table 2). All samples were stored in consumer packaging or polypropylene bags at laboratory temperature (21 ± 0.5 °C) in a dry place in the dark until sample preparation before analysis. Most samples originated from Asian and European countries (see Figure 1).

### 2.2. Sample Preparation—OTA Purification by Immunoaffinity Chromatography

All spice samples were properly homogenised using a laboratory homogenizer. The separation step was performed using a modified method according to Zimmerli and Dick [49] using immunoaffinity chromatography to increase both the selectivity and sensitivity of the method. The immunoaffinity chromatography uses immunoaffinity columns (IACs) operating specifically on the principle of antigen–antibody. The method consists of binding the antigen (OTA) by special anti-OTA antibodies anchored in the column. After the application of the washing solution, the other potentially interfering substances are removed from the column. The bound OTA is then released with acidified methanol from the antigen–antibody complex [49].

### 2.3. Chemicals and Apparatus

Methanol (CH_3_OH), acetonitrile (C_2_H_3_N), and chloroform (CHCl_3_) (all of HiPerSolv CHROMANORM gradient grade), formic acid 85% (HCOOH) pro-analysis (p.a.), orthophosphoric acid 85% (H_3_PO_4_) (HiPerSolv CHROMANORM), glacial acetic acid (CH₃COOH), di-sodium hydrogen phosphate anhydrous (Na_2_HPO_4_), sodium hydrogen carbonate (NaHCO_3_), sodium chloride (NaCl), and sodium hydroxide (NaOH) (all of AnalaR NORMAPUR) were purchased from VWR (Stříbrná Skalice, Czech Republic). All chemicals were stored at laboratory temperature 21 ± 0.5 °C. Analytical standard of OTA (*Petromyces albertensis*, ≥98%, HPLC) was purchased from VWR (Stříbrná Skalice, Czech Republic) and produced by Sigma-Aldrich spol. s r.o. (Prague, Czech Republic). The analytical standard was stored in a laboratory freezer at −20 °C. Immunoaffinity columns (IACs) OCHRAPREP^®^ were purchased from Jemo Trading spol. s r.o., Profood (Bratislava, Slovakia) and produced by R-Biopharm (Darmstadt, Germany). Nylon syringe filters (13 mm, 0.22 µm) produced by Labstore (Inverness, Highland, UK) and were purchased from HPST s.r.o. (Prague, Czech Republic).

All solutions were prepared in ultrapure water using a Milli-Q system (Millipore, Milford, MA, USA) (hereinafter referred to as ‘water’). The resistivity of ultrapure water was >18.2 MΩ.cm at 25 °C

A IKA A 10 basic homogeniser manufactured by IKA—WERKE GMBH & CO. KG (Staufen, Germany) was purchased from Fisher Scientific, spol. s r.o. (Pardubice, Czech Republic); an analytical balance KERN EW1500-2 manufactured by KERN & SOHN GmbH (Balingen, Germany) was purchased from Fisher Scientific, spol. s r.o. (Pardubice, Czech Republic); a Reax Multi shaker manufactured by Heidolph Instruments GmbH & Co. KG (Schwabach, Germany) was purchased from Fisher Scientific, spol. s r.o. (Pardubice, Czech Republic); and a laboratory centrifuge MPW 351e manufactured by MPW MED. Instruments (Warsaw, Poland) was purchased from Unimed Praha, spol. s r.o. (Prague, Czech Republic).

HPLC-FLD, Agilent 1260 Infinity II coupled to 1260 Infinity II Fluorescence Detector manufactured by Agilent (Santa Clara, CA, USA) was purchased from HPST s.r.o. (Prague, Czech Republic).

### 2.4. Solution Preparation

#### 2.4.1. 3% Solution of Sodium Hydrogen Carbonate (NaHCO_3_)

A total of 30 g of NaHCO_3_ was quantitatively transferred to a 1000 mL volumetric flask (hereinafter referred to as ‘flask’) and dissolved in a small amount of water. After dissolving the batch, the flask was made up with water.

#### 2.4.2. Phosphate Saline Buffer Containing 15% Methanol (PBS-15% Methanol)

PBS consists of two solutions: solution A (0.02 mol L^−1^ Na_2_HPO_4_ at pH 7.4) and solution B (0.29 mol L^−1^ NaCl). Solution A: A total of 1.42 g of NaHPO_4_ was quantitatively transferred to a 500 mL flask and dissolved in a small amount of water. After dissolving the batch, the flask was made up with water. The pH at 7.4 was adjusted with 85% H_3_PO_4_. Solution B: A total of 8.47 g of NaCl was quantitatively transferred to a 500 mL flask and dissolved in a small amount of water. After dissolving the batch, the flask was made up with water. PBS was obtained by mixing both prepared solutions A and B in a ratio of 1:1. PBS is stable for one year. PBS-15% methanol was obtained by mixing 850 mL of PBS and 150 mL of methanol.

#### 2.4.3. 3% Buffer Solution of Ortho-Phosphoric Acid (H_3_PO_4_) and Sodium Chloride (NaCl) at pH 1.6

A total of 116.9 g of NaCl was quantitatively transferred to a 1000 mL flask and dissolved in a small amount of water. After dissolving the batch, a total of 33.7 mL of H_3_PO_4_ was pipetted into the flask. The flask was made up with water. The pH at 1.6 was adjusted with NaOH. The solution is stable for six months.

#### 2.4.4. Elution Solution of Methanol (CH_3_OH) Acidified by Glacial Acetic Acid (CH_3_COOH)

A total of 2 mL of CH_3_COOH was pipetted into a 100 mL flask. The flask was made up with CH_3_OH.

All of these solutions were kept at 5 ± 0.5 °C. Before direct use, they were tempered at a laboratory temperature of 21 ± 0.5 °C.

#### 2.4.5. OTA Working Standard Solution at a Concentration of 40 µg L^−1^ (25 mL)

A total of 100 µL of OTA stock solution at concentration of 1000 µg L^−1^ was pipetted into a 25 mL flask. The flask was made up with CH_3_OH.

Stock and working standard solutions were kept at −20 ± 0.5 °C.

#### 2.4.6. Calibration OTA Standards

A total of six calibration OTA standards (0.10, 0.25, 0.50, 1.00, 2.00, and 4.0 ng mL^−1^) were prepared with a linear response on each day of the measurement from the working solution (40 µg L^−1^) by its dilution in the mobile phase (MP) in a ratio reaching the target concentration. The determination coefficient was 0.9999. A blank sample consisting of the mobile phase was also prepared fresh daily.

#### 2.4.7. Mobile Phase (MP)

The MP consisted of two solutions: solution A (acetonitrile:acetic acid, 99:1) and solvent B (water:acetic acid, 99:1). Solvents were used in ratio 40:60; A:B.

### 2.5. Workflow

#### 2.5.1. OTA Extraction

A total of 2 g of the sample was weighed into a polypropylene centrifuge tube (hereinafter referred to as the tube), 10 mL of buffer was added and left to shake using Vortex (1 min). The extraction step with 4 × 5 mL of chloroform was performed using Vortex (3 min) and a centrifuge (15 min; 3305× *g*; at laboratory temperature 21 ± 0.5 °C). The lower chloroform phase was collected into a glass vial and left to evaporate under nitrogen at 45 °C to dryness. The residue was dissolved in 5 mL of chloroform using Vortex (5 min). The dissolved residuum was transferred to a new tube. Extraction with 2 × 5 mL of 3% solution of sodium bicarbonate was performed using Vortex (3 min) and a centrifuge (5 min; 2000× *g*, at laboratory temperature 21 ± 0.5 °C) to achieve a compact thin layer between two phases. The upper aqueous bicarbonate phase was collected into a new tube in which 1 mL of chloroform and 0.5 mL of 85% formic acid had been prepared. The re-extraction of aqueous bicarbonate with 2 × 2 mL of chloroform was performed using the Vortex (3 min) and centrifuge (5 min; 2000× *g*, at laboratory temperature 21 ± 0.5 °C) to achieve a compact thin layer between two phases. The lower chloroform phase at the bottom of the tube was collected into a glass vial and left to evaporate to dryness under a nitrogen stream at 45 °C.

#### 2.5.2. OTA Separation

The residue was dissolved in 20 mL of PBS–methanol 15% using Vortex (5 min). A laboratory ultrasonic bath was used (10 min) to enhance the dissolution of the residue.

The IACs were brought to the laboratory temperature (at 21 ± 0.5 °C for approximately ½–1 h) and the buffer was released. A total of 20 mL of PBS–methanol 15% was transferred to the reservoir above the IAC and left to pass through IAC (at one drop per second; 2 mL min^−1^). The IACs were purified with 20 mL of water (at one drop per s) followed by brief air sieving (1–2 s). The elution of potential OTA was performed with 1.5 mL of methanol:acetic acid (98:2) into a small glass vial (at one drop per second) and followed by strong air sieving (30 s). The 1.5 mL eluate was evaporated under a nitrogen stream at 45 °C to dryness and kept in the laboratory fridge at 4 °C until analysis with HPLC-FLD. Before analysis, samples were dissolved in 0.5 mL of MP using an ultrasonic bath (5 min; 37 kHz; at laboratory temperature 21 ± 0.5 °C) and passed through a nylon syringe filter (13 mm, 0.22 µm) into a vial for HPLC.

#### 2.5.3. Analysis of Ochratoxin A in Spices by HPLC-FLD

**Figure 2:**
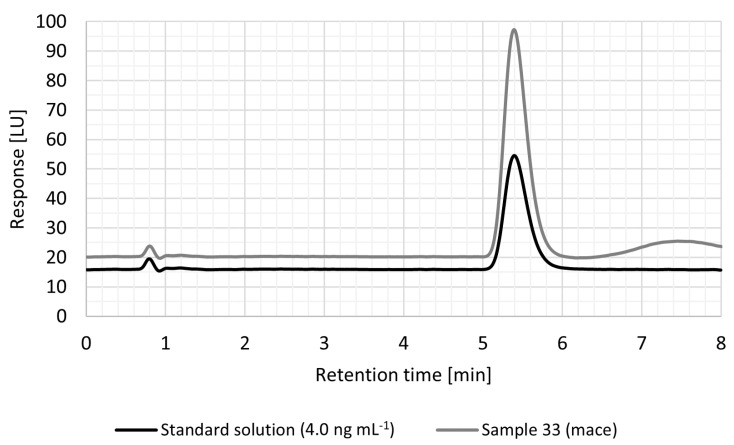
HPLC-FLD chromatogram showing peaks of OTA in spice sample 33 (mace) and standard solution (4.0 ng mL^−1^) at a retention time of 5.4 min.

HPLC-FLD was employed for the determination of OTA. The column (Kinetex C18, 2.6 µm, 100 Å, 50 × 21 mm) coupled with a security GuardTM column (Phenomenex C18, 4 × 2.0 mm) was purchased from Chromservis s.r.o. (Prague, Czech Republic) and were used and kept at 30 °C. The MP was set at a flow rate of 0.2 mL min^−1^. The injection volume was 8.0 µL. Fluorescence detection was performed at an excitation wavelength of 333 nm and an emission wavelength of 465 nm, PMT gain 18, attenuation 100 LU. Chromatography software Agilent OpenLab software was used to collect the chromatographic data. The method was validated. The limit of detection (LOD) was 0.03 ng g^−1^ and the limit of quantification (LOQ) was 0.1 ng g^−1^. The recovery of the method was verified using samples spiked with OTA. No reference material for the determination of OTA in spices was available during the period of this research. Therefore, the recovery was performed via spiked spice samples at OTA concentration levels of 0.5 and 2.0 ng g^−1^. OTA levels of 0.5 ng g^−1^ and 2.0 ng g^−1^ were added to the matrix before the extraction step, both concentrations in triplicate. The same concentrations were added after the separation step on immunoaffinity columns to the eluate, both concentrations in triplicate again. A total of 12 spiked samples were analysed for OTA. The recovery was determined for both concentration levels based on matrix effect—the ratio of the mean concentrations of samples with spiked matrix and samples with spiked eluate. The mean recovery was 74.2%, which fulfils the requirements of Regulation (EC) No. 401/2006 [50]. The repeatability standard deviation (RSD) was 0.76%. The mean measurement uncertainty was 4.03% including all kind of spices. OTA retention time was 5.4 min. The calibration curve consisted of six levels of concentrations (0.10, 0.25, 0.50, 1.00, 2.00, and 4.00 ng mL^−1^). All samples with a concentration outside the calibration curve were diluted or concentrated to reach value within the calibration curve. The chromatographs of OTA standard solution (4.00 ng mL^−1^) and one of the samples (33-mace) are shown in Figure 2.

## 3. Results

A total of 101 (31%) spice samples of 19 spice kinds were positive (exceeding LOQ of 0.1 ng g^−1^) for OTA (see Table 3). The concentrations of positive samples were in the range of 0.11 ng g^−1^ (for pink pepper) to 38–46 ng g^−1^ (for turmeric).

## 4. Discussion

### 4.1. Comparison of OTA Results in Spices with Other Relevant Studies in the World

To our knowledge, the set of spices analysed in this study has not been comprehensively analysed for OTA in other research papers, which makes it difficult to compare the whole dataset with the existing studies. Moreover, some types of spices included in this study such as asafoetida, black cumin, calamint, celery root, chervil, chives, citronella, dried dill tip, galangal root, green pepper, juniper, lemon peel, lovage, orange peel, pink pepper, Sichuan pepper, star anise, vanilla, white mustard, and wild garlic have never, or not recently, been analysed for the presence of OTA in other studies. The benefit of this study is certainly a positive OTA finding in some of these previously unanalysed spices such as lemon peel, orange peel, pink pepper, vanilla, and white mustard.

Therefore, we focused on evaluating our above-detection limit results in relation to the studies listed in Table 1. The comparability was possible with spices such as turmeric, liquorice, chilli, mace, ginger, cayenne pepper, sweet paprika, cumin, nutmeg, white pepper, black pepper, clove, and sumac.

To evaluate the general occurrence of OTA in given spices, we used categories from our previous study by Picková et al. [4]. These categories are based on the percentage of a total number of positive findings out of a total number of samples tested in a given spice based on recent relevant studies since 2015. These categories are: ‘no occurrence’ (0%), ‘rare occurrence’ (up to 5%), ‘low occurrence’ (up to 25%), ‘moderate occurrence’ (up to 50%), ‘high occurrence’ (up to 75%), and ‘very high occurrence’ (more than 75%).

OTA in turmeric was found to be of a ‘moderate occurrence’. Across all studies presented in Table 1, the average OTA concentration in turmeric was in the range of 1.89–162.00 ng g^−1^ [23,24,26,29,30,31,45] or was not detected at all [27]. In our study, the average concentration of 17.04 ng g^−1^ in turmeric fell within the range found in the literature. The greatest similarity of results could be observed with the average OTA concentration of 11.72 ng g^−1^ in the Polish study [45]. In our study, it was the only one spice kind with OTA concentration exceeding the EU limit of 15 ng g^−1^ [47]. Given the origin of our samples of turmeric in India, undoubtedly the largest producer of spices in the world [5], the average concentration of 17.04 ng g^−1^ does not seem to be as severe as the average concentration of 162.00 ng g^−1^ found in the Indian study [24].

OTA in liquorice was found to be of a ‘moderate occurrence’ [9]. Across all studies mentioned in Table 1, the average OTA concentration in liquorice was found in the range of 2.00–15.80 ng g^−1^ in only two studies [22,40]. In our study, liquorice with the average OTA concentration of 11.94 ng g^−1^ was within the range found in the literature and was nearly in line with the OTA concentration of 15.80 ng g^−1^ in the Czech study [40]. The EU limit of 20 ng g^−1^ for OTA was not exceeded in any of the liquorice samples [47].

OTA in chilli was found to be of a ‘moderate occurrence’ [4]. Across all of the studies mentioned in Table 1, the average OTA concentration was found in the range of 0.62–97.10 ng g^−1^ [16,17,19,21,23,25,26,29,30,31,33,37,39,40], but it was not detected in another study [32]. In our study, both average OTA concentrations of 7.50 ng g^−1^ in milled chilli and 1.43 ng g^−1^ in crushed chilli with seeds were within the range found in the literature. The average OTA concentration for milled chilli was very similar to the average OTA concentrations of 6.77 ng g^−1^ in Chinese [21], 7.70 ng g^−1^ in Lebanese [29], 7.15 ng g^−1^ in Malaysian [33], and 6.7 ng g^−1^ in Czech studies [40], while for crushed chilli with seeds to the OTA concentrations of 1.50 ng g^−1^ in the Ivory Coast study [17]. The EU limit of 20 ng g^−1^ for OTA was not exceeded in any of the chilli samples [47].

OTA in mace was found to be of a ‘high occurrence’ [4]. It is necessary to note that this statement was based on only one Indian study in which the average OTA concentration of 128 ng g^−1^ was found in mace [24]. In contrast, the average OTA concentration of 5.27 ng g^−1^ in mace was much lower in this study.

OTA in ginger was found to be of a ‘moderate occurrence’ [4]. Across all studies mentioned in Table 1, the average OTA concentration was found in the range of 0.22–82.80 ng g^−1^ [16,17,18,23], but it was not detected at all in other studies [29,30]. In our study, the average OTA concentration of 3.40 ng g^−1^ in ginger was in the range found in the literature. The greatest similarity was observed with the average OTA concentration of 3.75 ng g^−1^ found in the Nigerian study [18]. The EU limit of 15 ng g^−1^ for OTA was not exceeded in any of the ginger samples [47].

The average OTA concentration of 2.59 ng g^−1^ in cayenne pepper in our study was not in line with the average OTA concentration of 45.64 ng g^−1^ in the Polish study, which was the only study available for the comparison. The EU limit of 15 ng g^−1^ for OTA was not exceeded in any of the cayenne pepper samples [47].

OTA in paprika was found to be of a ‘high occurrence’ [4]. Across all studies mentioned in Table 1, the average OTA concentration was found in the range of 11.00–39.64 ng g^−1^ [19,29,30,40,42,46], but was not detected in another study [27]. In our study, the average OTA concentration of 2.26 ng g^−1^ in sweet paprika was not in the range found in the literature, as it was lower than the average OTA concentration of 11.00 ng g^−1^ in the Lebanese study [29]. The EU limit of 15 ng g^−1^ for OTA was not exceeded in any of the sweet paprika samples [47].

OTA in cumin was found to be of a ‘low occurrence’ [4]. Across all studies mentioned in Table 1, the average OTA concentration was found in the range of 0.63–20.4 ng g^−1^ [21,29,31,39], but it was not detected at all in another study [23]. In our study, the average OTA concentration of 0.46 ng g^−1^ in cumin was not within the range found in the literature as it was lower than the average OTA concentration of 0.63 ng g^−1^ in the Turkish study [39].

OTA in nutmeg was found to be of a ‘very high occurrence’ [4]. Across all studies mentioned in Table 1, the average OTA concentration was found in the range of 2.73–34.00 ng g^−1^ [29,30,40,44,45]. In our study, the average OTA concentration of 0.43 ng g^−1^ in nutmeg was not within the range found in the literature as it was lower than the average OTA concentration of 2.73 ng g^−1^ in the Polish study [45]. The EU limit of 15 ng g^−1^ for OTA was not exceeded in any of the nutmeg samples [47].

OTA in white pepper was found to be of a ‘low occurrence’ [4]. Across all studies mentioned in Table 1, the average OTA was found in the range of 3.30–29.41 ng g^−1^ in only two studies [15,45], but it was not detected at all in other studies [20,29,30,31,41]. In our study, the average OTA concentration of 0.36 ng g^−1^ in white pepper was not within the range found in the literature as it was lower than the average OTA concentration of 3.30 ng g^−1^ in the Cameroonian study [39]. The EU limit of 15 ng g^−1^ for OTA was not exceeded in any of the white pepper samples [47].

OTA in black pepper was found to be of a ‘moderate occurrence’ [4]. Across all studies mentioned in Table 2, the average OTA concentration was in the range of 0.34–155.00 ng g^−1^ [15,17,23,24,25,27,29,30,31,39,40,45] or was not detected at all [16,20,31,41,44]. In our study, the average OTA concentration of 0.31 ng g^−1^ in black pepper was not within the range found in the literature as it was found to be very similar, but slightly lower, than the OTA concentration of 0.0.34 ng g^−1^ in the Turkish study [39]. The EU limit of 15 ng g^−1^ for OTA was not exceeded in any of the black pepper samples [47].

OTA in cloves was found to be of a ‘none occurrence’ [4], however, the study by Pickova et al. [4] dealt with only the most recent publications concerning spices since 2015 [15,20,29,30]. There has been one case of a positive finding with the average OTA concentration of 0.48 ng g^−1^ in cloves in an older Poland study [45] with which our result is in agreement as the average OTA concentration of 0.29 ng g^−1^ was in cloves.

OTA in sumac was found to be of a ‘none occurrence’ [4]. This statement was based on only one study in Lebanon [29]. In contrast, our study provided a positive finding of OTA in sumac with an average concentration of 0.14 ng g^−1^, which can be considered a benefit of the study.

OTA was not found in the other spice kinds included in this study. Our under-detection limit results were in line with the statement of ‘none occurrence’ in cases of allspice [29,30], basil [29,44], bay leaf [29], mint [29], oregano [20,29,44], parsley [29], saffron [29], and thyme [29,44]. However, there is one older Polish study that contradicts this statement and thus our results, as it presented positive results for the presence of OTA in allspice, basil, oregano, and thyme [45].

### 4.2. Comparison of OTA Results in Spices with the Maximal Limits of the EU Legislation

Commission Regulation (EC) No. 1881/2006 [47] is one of the most extensive and stringent regulations setting maximum limits for certain contaminants including mycotoxins in foodstuffs, as amended, and is suitable for comparing the results obtained, especially because of its complexity with regard to spices. Moreover, all samples were purchased in the Czech Republic, which is one of the 27 Member States of the EU, therefore only the EU limits were considered. Results showed that only one sample (50-turmeric) was contaminated with OTA at a concentration exceeding the maximal limit set by the European Union. A comparison of OTA concentrations that have been found so far in regulated spices with the maximal limits of the EU legislation is presented in Table 4.

### 4.3. Proposal for New Maximum Limits for OTA in the EU and FAO/WHO Codex Alimentarius

The issue of OTA was also recently discussed in the meeting from 14 to 15 July 2021 at the Working Group for Agricultural Contaminants of the Directorate-General for Health and Food Safety, the European Commission. Amendments to the draft maximum levels for OTA in food for which there are currently no limits and the draft maximum limits for spices for which there are currently limits are currently under discussion and consideration (see Table 5) [48].

The issue of OTA was also recently discussed in the report of the 14th Session of the Codex Committee on Contaminants in Foods by the Codex Alimentarius Commission (virtual) 3–7 and 13 May 2021. They discussed the maximum limits for OTA in nutmeg, dried chilli and paprika, ginger, pepper, and turmeric for comments and consideration by the Session of the Codex Committee on Contaminants in Foods in the year 2022. Maximum limits of 15–20 ng g^−1^ for OTA in spices should be established [52].

### 4.4. The Occurrence of OTA in Spices on Data by RASFF (2016–2021)

**Figure 3:**
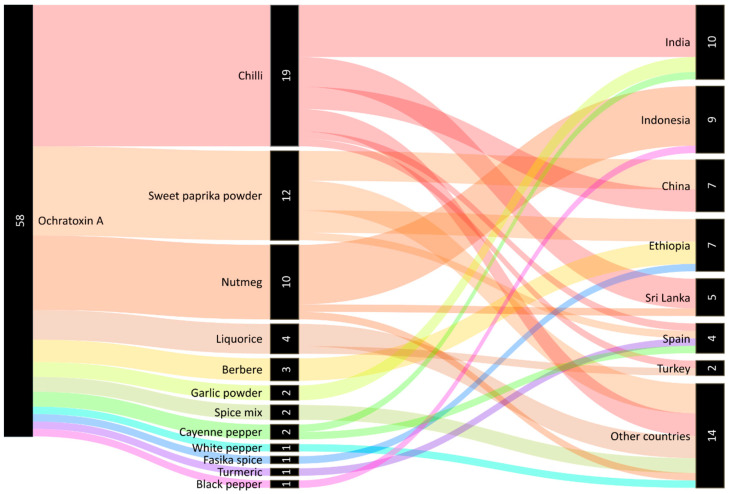
Notifications of ochratoxin A in spices by the Rapid Alert System for Food and Feed (RASFF) since 2016 (current to the 20 July 2021). Notes: The category ‘Other countries’ includes all countries of origin with only one notification for spices: Azerbaijan, Bangladesh, France, Germany, Hungary, Italy, Lebanon, Peru, Portugal, Thailand, Ukraine, the United Kingdom, and Vietnam, or notification of unknown origin. Processed according to the RASFF database [53] using The Sankey Diagram Generator online tool and vector graphics editor Inkscape 0.92.

Rapid Alert System for Food and Feed (RASFF) is a key tool ensuring food safety in the context of the EU and enables one to orientate oneself in the issue of OTA occurrence in various foods including spices. Notifications reporting the presence of OTA in spices are also valuable information for completing the idea of the current state of distributed spices. Based on the RASFF database, a total of 58 OTA notifications have been related to spices since 2016 (see Figure 3). The most prevalent notifications concerned OTA in chilli (33%), sweet paprika powder (21%), and nutmeg (17%). Most OTA notifications originated in India (17%, mostly chilli) and Indonesia (16%, mostly nutmeg).

## 5. Conclusions

Human dietary exposure to OTA from foodstuffs is very common. Despite various effective methods for OTA mitigation and the reduction of possible health risks of OTA in foodstuffs, OTA is still a persistent problem. Although spices are not among the main sources of daily OTA intake in humans, they may contribute significantly to the co-exposure with major OTA sources such as cereals, wine, pork meat, and coffee. This may result in an additive effect and thus an increase in OTA toxicity. The significance of this study lies in the analysis of a large number of types of spices for OTA, focusing only on single-species spices, not mixtures of spices. In this study, the analysis of 54 single-kind species showed a total of 19 (35%) OTA-positive spice kinds, meaning that at least one sample of a given spice kind exceeded LOQ by its concentration. Among these OTA-positive spice kinds were turmeric, liquorice root, chilli milled, mace, ginger, cayenne pepper, sweet paprika, chilli crushed with seeds, vanilla, orange peel, cumin, nutmeg, white mustard, white pepper, black pepper, clove, lemon peel, sumac, and pink pepper. This study therefore demonstrates that the Czech population is exposed to OTA through various contaminated single-kind spices available on the Czech market.

As can be seen, the spice kinds with OTA-positive findings included regulated spice kinds but also those for which regulation has not yet been set, namely mace, vanilla, orange peel, cumin, white mustard, cloves, lemon peel, sumac, and basil. Fortunately, promising discussions are already taking place in the European Commission, in which, among other things, a limit for ‘all spices’ has been proposed at 15 ng g^−1^, but has not been adopted yet. Taking into consideration this proposed limit for all hitherto unregulated spices, none of the analysed spice samples exceeded this value in this study.

In terms of public health protection, where food safety is an important preventive component, it is necessary to regulate various mycotoxin contents in various spices. Hence, our future research will focus not only on OTA monitoring, but also on the other mycotoxins in spices, as it will be important to verify the mycotoxin intake from this commodity.

## Figures and Tables

**Table 1:** Overview of studies dealing with the contamination of spices with OTA from a global perspective.

Country	Spices	*n*+/*n*	*n*+%	Mean (ng g^−1^)	Range Min–Max (ng g^−1^)	LOQ (ng g^−1^)	References
Africa							
Cameroon	Black pepper	2/20	10	1.53	1.15–1.91	1.00	[15]
	Cloves	0/40	0	-	-	1.00	
	White pepper	8/20	40	3.30	1.81–4.89	1.00	
Ivory Coast	Black pepper	0/30	0	-	-	0.20	[16]
	Chilli	25/30	83	68.97 ^↑^	0.04–907.57	0.20	
	Ginger	15/30	50	0.22	0.04–0.56	0.20	
	Black pepper	7/12	58	4.56	0.27–13.95	0.16	[17]
	Chilli	4/12	33	1.50	0.23–4.45	0.16	
	Dawadawa	2/12	17	1.40	1.26–1.55	0.16	
	Ginger	3/12	25	0.22	0.17–0.31	0.16	
Nigeria	Ginger	57/120	48	3.75	0.17–12.02	0.30	[18]
South Africa	Chilli	2/18	11	16.00	7.00–25.00	4.20	[19]
	Fruit chutney spices	1/4	25	6.00 *	6.00 *	4.20	
	Onion	0/8	0	-	-	4.20	
	Paprika	1/7	14	11.00 *	11.00 *	4.20	
	Vegetable spice	0/1	0	-	-	4.20	
America							
Brazil	Black pepper	0/15	0	-	-	N/S	[20]
	Chilli	0/15	0	-	-	N/S	
	Cinnamon	0/13	0	-	-	N/S	
	Cloves	0/12	0	-	-	N/S	
	Fennel	0/15	0	-	-	N/S	
	Oregano	0/12	0	-	-	N/S	
	Rosemary	0/15	0	-	-	N/S	
	White pepper	0/15	0	-	-	N/S	
Asia							
China	Aniseed	5/80	6	0.73	N/S	0.50	[21]
	Chilli	15/80	19	6.77	N/S	0.50	
	Cinnamon	4/80	5	1.10	N/S	0.50	
	Cumin	5/29	17	1.46	N/S	0.50	
	Curry powder	5/11	46	2.44	N/S	0.50	
	Fennel	0/40	0	-	-	0.50	
	Pepper	0/80	0	-	-	0.50	
	Prickly ash	8/80	10	3.17	N/S	0.50	
	Liquorice	2/31	6	2.00	0.06–3.93		[22]
India	Chilli	40/55	73	97.10 ^↑^	N/S	N/S	[23]
	Black pepper	33/42	79	154.10 ^↑^	N/S	N/S	
	Caraway	12/25	48	63.20 ^↗^	N/S	N/S	
	Coriander	9/30	30	47.60 ^↗^	N/S	N/S	
	Cumin	0/28	0	-	-	N/S	
	Fennel	14/25	56	98.10 ^↗^	N/S	N/S	
	Fenugreek	18/35	51	83.20 ^↗^	N/S	N/S	
	Ginger	20/36	56	82.80 ^↑^	N/S	N/S	
	Turmeric	20/35	57	125.90 ^↑^	N/S	N/S	
	Black pepper	31/55	56	155.00	N/S	N/S	[24]
	Cardamom	11/32	34	68.00	N/S	N/S	
	Fennel	8/35	23	10.00	N/S	N/S	
	Mace	18/30	60	128.00	N/S	N/S	
	Turmeric	21/42	50	162.00	N/S	N/S	
Indonesia	Chilli	3/6	50	44.87 ^↑^	23.70–84.60	1.77	[25]
Iran	Black pepper	10/23	43	3.31	0.70–7.64	0.06	[26]
	Cinnamon	8/23	35	5.46	0.45–16.10	0.06	
	Chilli	4/23	17	5.66	0.56–18.64	0.06	
	Turmeric	7/23	30	2.77	0.60–8.49	0.06	
	Black pepper	20/20	100	49.29	15.91–197.64	1.23	[27]
	Cinnamon	2/20	10	18.5	0.70–139.44	1.23	
	Chilli	0/20	0	-	-	1.23	
	Turmeric	0/20	0	-	-	1.23	
Korea	Chilli	6/56	11	2.38	4.51 ^M^	0.31	[28]
Lebanon	Allspice	0/3	0	-	-	1.50	[29]
	Anise	1/3	33	2.60 *	2.60 *	1.50	
	Basil	0/2	0	-	-	1.50	
	Bay leaf	0/2	0	-	-	1.50	
	Black pepper	1/4	25	2.30 *	2.30 *	1.50	
	Caraway	0/2	0	-	-	1.50	
	Cardamom	0/4	0	-	-	1.50	
	Chilli	2/7	29	7.70	N/S	1.50	
	Cinnamon	0/3	0	-	-	1.50	
	Cloves	0/2	0	-	-	1.50	
	Coriander	0/2	0	-	-	1.50	
	Cumin	1/5	20	3.50 *	3.50 *	1.50	
	Fennel	0/2	0	-	-	1.50	
	Fenugreek	0/4	0	-	-	1.50	
	Garlic	1/2	50	5.10 *	5.10 *	1.50	
	Ginger	0/3	0	-	-	1.50	
	Marjoram	1/2	50	0.75 *	0.75 *	1.50	
	Mint	0/3	0	-	-	1.50	
	Nutmeg	1/2	50	33.90 * ^↑^	33.90 *	1.50	
	Onion	0/4	0	-	-	1.50	
	Oregano	0/3	0	-	-	1.50	
	Paprika	2/3	67	11.40	N/S	1.50	
	Parsley	0/1	0	-	-	1.50	
	Rosemary	1/2	50	0.75 *	0.75 *	1.50	
	Saffron	0/1	0	-	-	1.50	
	Sage	1/3	33	4.20 *	4.20 *	1.50	
	Sumac	0/2	0	-	-	1.50	
	Thyme	0/3	0	-	-	1.50	
	Turmeric	1/2	50	2.40 *	2.40 *	1.50	
	White pepper	0/2	0	-	-	1.50	
	Allspice	N/S	ND	-	-	1.50	[30]
	Anise	N/S	D	2.6	N/S	1.50	
	Black pepper	N/S	D	2.30	N/S	1.50	
	Cardamom	N/S	ND	-	-	1.50	
	Caraway	N/S	ND	-	-	1.50	
	Cinnamon	N/S	ND	-	-	1.50	
	Cloves	N/S	ND	-	-	1.50	
	Coriander	N/S	ND	-	-	1.50	
	Cumin	N/S	D	3.50	N/S	1.50	
	Fennel	N/S	ND	-	-	1.50	
	Garlic powder	N/S	ND	-	-	1.50	
	Ginger	N/S	ND	-	-	1.50	
	Nutmeg	N/S	D	34.00 ^↑^	N/S	1.50	
	Onion powder	N/S	ND	-	-	1.50	
	Paprika	N/S	D	11.40	N/S	1.50	
	Red chilli	N/S	D	7.70	N/S	1.50	
	Turmeric	N/S	D	2.40	N/S	1.50	
	White pepper	N/S	ND	-	-	1.50	
Malaysia	Coriander	1/1	100	0.91 *	0.91 *	0.33	[31]
	Cumin	1/2	50	20.40 * ^↗^	20.40 *	0.33	
	Curry	8/8	100	2.36	0.14–9.59	0.33	
	Chilli	1/2	50	0.62 *	0.62 *	0.33	
	Fennel	1/2	50	1.26 *	1.26 *	0.33	
	Black pepper	0/1	0	-	-	0.33	
	Turmeric	2/2	100	1.89	0.20–3.58	0.33	
	White pepper	0/1	0	-	-	0.33	
	Chilli	0/10	0	-	-	0.30	[32]
	Chilli	65/80	81	7.15	0.20–101.20	0.06	[33]
Pakistan	Chilli crushed, restaurant	14/28	50	19.80	48.70 ^M^	0.18	[34]
	Chilli powdered, restaurant	12/29	41	22.90 ^↑^	58.10 ^M^	0.18	
	Chilli crushed, open market	11/29	38	16.90	54.30 ^M^	0.18	
	Chilli powdered, open market	13/34	38	21.40 ^↑^	64.50 ^M^	0.18	
	Chilli	99/242	41	N/S	120.90 ^M^	0.30	[35]
Saudi Arabia	Cardamom	38/80	48	60.14 ^↗^	30.00–78.00	3.33	[36]
Sri Lanka	Chilli flakes	13/26	50	4.90	15.00 ^M^	N/S	[37]
	Chilli pods	2/18	11	N/S	5.30 ^M^	N/S	
	Red chilli powder	20/42	48	16.00	282.00 ^M^	N/S	
	Black pepper	N/S	D	N/S	79.00 ^M ↑^	N/S	[38]
	Chilli	35/86	41	N/S	282.00 ^M^	N/S	
Turkey	Black pepper	4/23	17	0.34	3.48 ^M^	0.19	[39]
	Cumin	1/19	5	0.63 *	0.63 *	0.19	
	Red chilli flakes	18/24	75	12.34	53.04 ^M^	0.19	
	Red chilli powder	12/22	55	13.46	98.20 ^M^	0.19	
Europe							
Czech Republic	Black pepper	11/12	92	0.83	2.82 ^M^	0.20	[40]
	Caraway	2/12	17	0.19	0.71 ^M^	0.20	
	Chilli pepper dried	11/12	92	6.70	32.70 ^M^	0.20	
	Coriander seed	4/12	33	0.46	1.96 ^M^	0.20	
	Fiery paprika powder	12/12	100	19.00	5.5–91.80	0.20	
	Ginger root dried	7/12	58	2.04	12.70 ^M^	0.20	
	Liquorice	12/12	100	15.80	3.8–36.70	0.20	
	Nutmeg	12/12	100	8.70	0.3–60.70	0.20	
	Sweet paprika powder	12/12	100	16.00	1.1–38.40	0.20	
Hungary	Black pepper	0/6	0	-	-	0.60	[41]
	Chilli	1/5	20	2.1 *	2.1 *	0.60	
	Red pepper, ground	32/70	46	N/S	0.4–66.2	0.60	
	White pepper	0/5	0	-	-	0.60	
Italy	Paprika	17/31	55	39.64 ^↑^	0.11–177.40	0.22	[42]
	Chilli	15/25	60	N/S	2.16–16.35	2.13	[43]
	Pepper	4/30	13.3	N/S	1.61–15.85	2.61	
Latvia	Basil	0/50	0	-	-	2.40	[44]
	Black pepper	0/50	0	-	-	1.50	
	Nutmeg	N/S	D	N/S	14.00 *	1.50	
	Oregano	0/50	0	-	-	2.40	
	Thyme	0/50	0	-	-	2.40	
Poland	Allspice	1/5	20	0.20 *	0.20 *	0.30	[45]
	Basil	1/3	33	0.05 *	0.05 *	0.30	
	Black pepper, grain	4/4	100	23.57 ^↑^	N/S	0.30	
	Black pepper, ground	4/5	80	9.46	N/S	0.30	
	Cayenne pepper	5/8	63	45.64 ^↑^	N/S	0.30	
	Cinnamon	3/4	75	2.14	N/S	0.30	
	Cloves	1/2	50	0.48 *	0.48 *	0.30	
	Curry	5/5	100	19.01 ^↗^	N/S	0.30	
	Garlic	2/3	67	0.11	N/S	0.30	
	Marjoram	4/5	80	7.13	N/S	0.30	
	Nutmeg	2/5	40	2.73	N/S	0.30	
	Oregano, whole	1/2	50	9.38 *	9.38 *	0.30	
	Oregano, crushed	2/4	50	22.12 ^↗^	N/S	0.30	
	Rosemary	1/2	50	5.07 *	5.07 *	0.30	
	Tarragon	1/1	100	6.98 *	6.98 *	0.30	
	Thyme	3/3	100	15.59 ^↗^	N/S	0.30	
	Turmeric	1/1	100	11.72 *	11.72 *	0.30	
	White pepper	6/7	86	29.41 ^↑^	N/S	0.30	
Spain	Chilli	35/35	100	N/S	0.62–44.60	0.10	[46]
	Paprika	63/64	98	N/S	281.00 ^M^	0.10	

Note: n: number of samples; n+: number of positive samples: n+%: per cent of positive samples; *: the only measured concentration; ^M^: the maximum concentration (the whole range is not known); -: no data; N/S: not specified; D: detected (the quantity of positive samples is not known); ND: not detected; ↑: the average OTA concentrations in regulated spices exceeding the relevant European Union limits EC No. 1881/2006 as in force [47]; ↗: the average OTA concentrations exceeding the limit of 15 ng g^−1^, which is currently proposed for ‘all spices’ by the European Commission [48].

**Table 2:** The characterisation of spice samples.

No.	Spices	Latin Name	Form	Country of Origin
1	Allspice	*Pigmenta officinalis* Lindl.	milled	Mexico
2	Anise	*Pimpinella anisum* L.	whole	Egypt
3	Asafoetida *	*Ferula assa-foetida* L.	milled	India
4	Basil	*Ocimum basilicum* L.	scrubbed	Egypt
5	Bay leaf	*Laurus nobilis* L.	milled	Turkey
6	Black cumin *	*Nigella sativa* L.	whole	India
7	Black pepper	*Piper nigrum* L.	milled	Spain
8	Calamint *	*Saturea hortensis* L.	scrubbed	Germany
9	Caraway	*Carum carvi* L.	milled	Czech Republic
10	Cardamom	*Elateria cardamomum* L.	milled	Guatemala
11	Cayenne pepper	*Capsicum frutescens* L.	milled	Indonesia
12	Celery root *	*Apium graveolens* L.	whole	Czech Republic
13	Chervil *	*Anthriscus cerefolium* (L.) Hoffm.	scrubbed	Germany
14	Chilli crushed with seeds	*Capsicum frutescens L.*	crushed	Thailand
15	Chilli milled	*Capsicum frutescens* L.	milled	India
16	Chives *	*Allium schoenoprasum* L.	chopped	China
17	Cinnamon	*Cinnamomum burmannii* (Nees & Th. Nees) Nees ex Blume	milled	Indonesia
18	Citronella *	*Cymbopogon citratus* (DC- ex Nees) Stapf	cut	Albania
19	Clove	*Eugenia caryophyllata* L.	milled	Madagascar
20	Coriander	*Coriandrum sativum* L.	milled	Czech Republic
21	Cumin	*Cuminum cyminum* L.	milled	India
22	Dried dill tip *	*Anetum graveolens* L.	chopped	Czech Republic
23	Fennel	*Foeniculum vulgare* Mill.	whole	Egypt
24	Fenugreek	*Trigonella foenum-graecum* L.	milled	India
25	Galangal root *	*Alpinia glanga* (L.) Wild.	milled	China
26	Garlic	*Allium sativum* L.	granulated	China
27	Ginger	*Zingiber officinale* Roscoe	milled	Peru
28	Green pepper *	*Piper nigrum* L.	milled	India
29	Juniper *	*Juniperus communis* L.	milled	Pakistan
30	Lemon peel *	*Citrus limon* (L.) Burm. f.	milled	Spain
31	Liquorice root	*Glycyrhiza glabra* L.	crushed	China
32	Lovage *	*Levisticum officinale* W.D.J. Koch	cut	Poland
33	Mace	*Myristica fragrans* Houtt.	milled	Indonesia
34	Marjoram	*Majorana hortensis* L.	scrubbed	Egypt
35	Mint	*Mentha piperita* L.	milled	Egypt
36	Nutmeg	*Myristica fragrans* Houtt.	milled	Indonesia
37	Orange peel *	*Citrus aurantium* L.	milled	Spain
38	Oregano	*Origanum vulgare* L.	cut	Turkey
39	Parsley	*Petroselinum sativum* Hoffm.	chopped	Poland
40	Pink pepper *	*Schinus terebinthifolius* Raddi	whole	Brazil
41	Rosemary	*Rosmarinus officinalis* L.	cut	Morocco
42	Saffron	*Crocus sativus* L.	whole	Spain
43	Sage	*Salvia officinalis* L.	scrubbed	Germany
44	Sichuan pepper *	*Zanthoxylum piperitum* (L.) DC.	whole	China
45	Star anise *	*Illicium verum* Hook. f.	milled	India
46	Sumac	*Rhus coriaria* L.	milled	Turkey
47	Sweet paprika	*Capsicum annuum* L.	milled	Hungary
48	Tarragon cut	*Artemisia dracunculus* L.	cut	Poland
49	Thyme	*Thymus vulgaris* L.	whole	Poland
50	Turmeric	*Curcuma longa* L.	milled	India
51	Vanilla *	*Vanilla planifolia* Jacks. Ex Andrews	milled	Tahiti
52	White pepper	*Piper nigrum* L.	milled	Vietnam
53	White mustard *	*Sinapis alba* L.	milled	Ukraine
54	Wild garlic *	*Allium usrinum* L.	cut	Bulgaria

Notes: *: spices in which OTA has never (or not recently) been studied according to the available literature (see Table 1).

**Table 3:** The concentrations of OTA in a total of 20 positive single-kind spices available on the Czech market.

No.	Kind of Spice	Incidence *n*+/*n*	Mean ± SD (ng g^−1^)	Median (ng g^−1^)	95th Perc. (ng g^−1^)	Range Min–Ma X (ng g^−1^)
50	Turmeric	6/6	19.82 ± 11.93	17.04	36.01	5.13–38.46
31	Liquorice root	6/6	11.94 ± 3.27	12.10	16.18	7.57–17.42
15	Chilli milled	6/6	7.50 ± 1.34	7.78	8.96	5.28–9.27
33	Mace	6/6	5.27 ± 0.83	5.25	6.22	3.94–6.33
27	Ginger	6/6	3.40 ± 0.48	3.46	3.91	2.18–3.93
11	Cayenne pepper	6/6	2.59 ± 0.61	2.71	3.21	1.67–3.27
47	Sweet paprika	6/6	2.26 ± 0.60	1.99	3.06	1.73–3.12
14	Chilli crushed with seeds	6/6	1.43 ± 0.48	1.41	1.98	0.82–2.03
51	Vanilla	6/6	1.42 ± 0.33	1.49	1.72	0.82–1.74
37	Orange peel	6/6	1.04 ± 0.30	1.04	1.41	0.63–1.47
21	Cumin	5/6	0.46 ± 0.27	0.55	0.70	<LOQ–0.72
36	Nutmeg	5/6	0.43 ± 0.30	0.49	0.78	<LOQ–0.84
53	White mustard	5/6	0.38 ± 0.30	0.32	0.76	<LOQ–0.79
52	White pepper	5/6	0.36 ± 0.23	0.37	0.61	<LOQ–0.62
7	Black pepper	5/6	0.31 ± 0.20	0.37	0.52	<LOQ–0.53
19	Clove	5/6	0.29 ± 0.18	0.33	0.48	<LOQ–0.50
30	Lemon peel	5/6	0.18 ± 0.12	0.18	0.32	<LOQ–0.36
46	Sumac	5/6	0.14 ± 0.08	0.14	0.24	<LOQ–0.26
40	Pink pepper	1/6	0.11 *	0.11 *	-	<LOQ–0.11

Note: n: number of samples; n+ = positive samples > LOQ = 0.10 ng g^−1^; SD = standard deviation; 95th perc = 95% percentile; *: the only one positive sample; left censored data: samples that contained OTA levels below LOQ were assigned a value 0 ng g^−1^ for statistical processing (<LOQ = 0 ng g^−1^)—the lower bound approach (LB) [51].

**Table 4:** The concentrations of OTA in positive single-kind spices available on the Czech market and comparison with the European Union legislation.

Number of Sample	Kind of Spice	OTA Concentration ^1^ (ng g^−1^)	EU Limits ^2^ (ng g^−1^)
50	Turmeric	19.82 ^3^	15
31	Liquorice root	11.94	20/80 ^4^
15	Chilli milled	7.50	20
27	Ginger	3.40	15
11	Cayenne pepper	2.59	20
47	Sweet paprika	2.26	15
14	Chilli crushed with seeds	1.43	20
36	Nutmeg	0.43	15
52	White pepper	0.36	15
7	Black pepper	0.31	15
40	Pink pepper	0.11	15

^1^ Positive samples are all samples with concentrations exceeding the limit of quantification of 0.13 ng g^−1^; ^2^ EU limits refer to the maximum levels of OTA in spices under the European Union—Regulation No. 1881/2006 as in force [47]; ^3^ OTA concentration exceeding the maximum permitted limit set by the European Union legislation; ^4^ The maximum limit of OTA of 20 ng g^−1^ is valid for ‘*Liquorice root*, *an ingredient for herbal infusion*’. The maximum limit of OTA of 80 ng g^−1^ is valid for ‘*Liquorice extract for use in food in particular beverages and confectionary*’ provided that it is pure and an undiluted extract is obtained whereby 1 kg of extract is obtained from 3 to 4 kg of liquorice root [47].

**Table 5:** The draft proposal of the maximum limits of OTA in spices.

Food	Proposal of Maximum Limits (ng g^−1^)
All spices including dried spices except *Capsicum* spp.	15
*Capsicum* spp. (dried fruits, whole or ground, including chilli, ground chilli, cayenne pepper and red pepper—paprika)	20
Mixtures of spices	15

Processed according to [48].

## Data Availability

All data are available from the corresponding authors.
